# Longitudinal Changes in Semantic Concreteness in Semantic Variant Primary Progressive Aphasia (svPPA)

**DOI:** 10.1523/ENEURO.0197-18.2018

**Published:** 2018-01-10

**Authors:** Katheryn A. Q. Cousins, Sharon Ash, Christopher A. Olm, Murray Grossman

**Affiliations:** 1 Department of Neurology and Penn Frontotemporal Degeneration Center; 2 Department of Radiology and Penn Image Computing and Science Laboratory, University of Pennsylvania, Philadelphia, PA 19104-4283

**Keywords:** concreteness, frontotemporal dementia, longitudinal, semantic memory, semantic variant primary progressive aphasia, ventral temporal lobe

## Abstract

This study examines longitudinal changes in the concreteness of nouns produced by human patients with semantic variant primary progressive aphasia (svPPA). Cross-sectional studies show that patients with svPPA demonstrate severe loss of concrete noun knowledge linked to atrophy of the left ventral temporal lobe. It is unknown how disease spread and duration affect the magnitude of the concreteness impairment in svPPA. We evaluate longitudinal spoken production of concrete nouns in svPPA, and relate this to changes in longitudinal MRI measures of gray matter (GM). Noun concreteness in svPPA is compared to that of behavioral variant frontotemporal dementia (bvFTD) patients, who typically demonstrate highly concrete speech. We elicited naturalistic speech samples at two time points (time 1 and time 2) in patients with svPPA (*n* = 11) and bvFTD (*n* = 15) through descriptions of the Cookie Theft picture and evaluated each spoken noun for concreteness. Compared to bvFTD patients whose noun production remained highly concrete throughout the testing period, mixed-effects models revealed that noun concreteness significantly decreased as disease progressed in svPPA. We also measured longitudinal changes to GM in a subset of svPPA patients (*n* = 7), who showed significant decline in the left and right temporal and frontal regions. Regression analyses revealed that longitudinal GM atrophy in the right fusiform and parahippocampal gyri and the left superior temporal gyrus was related to decreasing noun concreteness. These results suggest that progressive atrophy of the ventral temporal lobe in svPPA contributes to declining concrete noun production over time.

## Significance Statement

Semantic memory is the generalizable knowledge of the world around us, including objects, concepts, and words. It is profoundly impaired in a rare form of frontotemporal dementia (FTD), semantic variant primary progressive aphasia (svPPA). The specific impairment of concrete concept knowledge in svPPA, compared to abstract concept knowledge, provides important neurolinguistic understanding of how we process word meaning. Yet, almost no studies to date have related longitudinal cognitive changes to progressive neurodegeneration in svPPA. This study is the first to relate progressive atrophy in svPPA to longitudinal semantic changes; we find that progressive gray matter (GM) atrophy in right ventral temporal regions is associated with declining production of concrete nouns in svPPA.

## Introduction

Patients with semantic variant primary progressive aphasia (svPPA) suffer from a profound loss of lexical and conceptual knowledge associated with atrophy to the left anterior and ventral temporal lobe (Hodges and Patterson, 2007; Landin-Romero et al., 2016). Semantic knowledge in svPPA is not equally impaired across all word categories (Pulvermüller et al., 2010), and some studies report that svPPA patients demonstrate a more severe deficit for concrete than abstract words, a phenomenon known as “the reversal of the concreteness effect (CE)” (Breedin et al., 1994; Catricalà et al., 2014; Cousins et al., 2017; Joubert et al., 2017). This loss of concrete concept knowledge in svPPA is thought to be due in part to deterioration of the bilateral ventral temporal lobe, important for the representation and individuation of visual features of concrete object concepts (Lambon Ralph et al., 2010; Bonner and Price, 2013; Clarke and Tyler, 2014, 2015; Bonner et al., 2016; Shebani et al., 2017). By comparison, inferior frontal and superior temporal regions associated with abstract noun processing tend to be spared in svPPA until late stage disease (Sabsevitz et al., 2005; Wang et al., 2010; Binder and Desai, 2011). However, the reversal of CE is not present for all svPPA patients, and other cross-sectional studies find equivalent or even worse comprehension for abstract compared to concrete words in svPPA (Jefferies et al., 2009; Hoffman et al., 2013).

One possible source of variability in semantic impairment is heterogenous laterality, spread and progression of disease in svPPA (Grossman and Ash, 2004; Bright et al., 2008; Brambati et al., 2009; Kumfor et al., 2016). Anatomic longitudinal and cross-sectional studies of gray matter (GM) degradation show that early atrophy in the left anterior temporal lobe is a consistent feature in svPPA, although some patients show right laterality (Brambati et al., 2009; Kumfor et al., 2016). Early progression of disease tends to spread posteriorly across the left temporal lobe and to the right temporal lobe, encompassing much of the bilateral visual association cortex that is important for concrete noun representations (Bright et al., 2008; Brambati et al., 2009). By comparison, regions that have been linked to abstract noun and general language processing, including the left inferior frontal gyrus and left posterior superior temporal lobe, are typically preserved until later stages of disease (Bright et al., 2008; Rogalski et al., 2011). Thus, the presence and magnitude of the reversal or CE in svPPA may depend on the progression and stage of disease.

To date, there are very few longitudinal studies in svPPA that would shed light on the relationship between spreading atrophy and changes in concrete noun knowledge. Of the two studies we know of that examine longitudinal changes in concrete semantics in svPPA, evidence is sparse and conflicting: one case study finds that reversal of CE in an svPPA patient is most severe early on and decreases with time (Macoir, 2009), while another finds that impairment for concrete visual words becomes more severe with time (Hoffman et al., 2012). Because neither study directly examines how progressive neural atrophy related to changes in concrete knowledge, the basis for the observed differences in reversal of CE in svPPA remains unclear.

To understand how disease progression affects semantic impairment in svPPA, we conducted a longitudinal study of noun production in patients with mild to moderate svPPA. We obtained naturalistic, semi-structured speech samples from patients using the Cookie Theft picture description task (Goodglass and Kaplan, 1983) at two time points. We calculated the average concreteness of the nouns produced (Brysbaert et al., 2014) and compared longitudinal changes in concrete noun production in svPPA to patients with behavioral variant frontotemporal dementia (bvFTD). Disease in bvFTD is centered in the frontal lobe and is associated with executive dysfunction and personality changes, such as apathetic or disinhibited behavior (Piguet et al., 2011; Rascovsky et al., 2011; O’Connor et al., 2017). Compared with svPPA, most bvFTD patients have relatively spared medial and lateral temporal lobe integrity (Rosen et al., 2002), although temporal lobe atrophy may be apparent with later disease progression (Seeley et al., 2008; Landin-Romero et al., 2017). Patients with bvFTD also have relatively preserved concrete noun knowledge (Villamizar Caycedo et al., 2009), while subtle impairments for language and abstract noun knowledge have been associated with their executive dysfunction and frontal lobe atrophy (Charles et al., 2014; Cousins et al., 2016, 2017; Hardy et al., 2016). Thus, we predict noun production in bvFTD to be highly concrete and to become less abstract with time. Finally, we obtained longitudinal MR imaging in svPPA patients, and related changes in concrete noun production to changes in GM.

## Materials and Methods

### Subjects

Participants were 11 svPPA patients (seven female) and 15 bvFTD patients (four female), diagnosed by board-certified neurologists according to published criteria (Gorno-Tempini et al., 2011; Rascovsky et al., 2011). A total of 10 of 11 svPPA patients also demonstrated mild behavior and personality changes, such as compulsive ritualistic behaviors and disinhibition, which commonly co-occur with svPPA and have been associated with their right temporal lobe atrophy (Hodges and Patterson, 2007; Kamminga et al., 2015). Scores of apathy, disinhibition, and language were also available for a subset of patients (Table 1) from the Philadelphia brief assessment of cognition (PBAC; Libon et al., 2011). A repeated measures ANOVA of apathy showed a main effect of group (*F*_(19)_ = 6.6, *p* < 0.05), and a trend for group X session interaction (*F*_(14)_ = 4.34, *p* = 0.056), indicating that bvFTD patients were more apathetic than svPPA patients. No effects were significant for Disinhibition. A repeated measures ANOVA of language score showed a main effect of group (*F*_(18)_ = 27.67, *p* < 0.001), but no effect of testing session (all *p* > 0.1) or a group X session interaction (all *p* > 0.1).

**Table 1.** Demographic and neuropsychological data for all subjects

	bvFTD			svPPA			*t* tests	
	M	SD	*n*	M	SD	*n*	*t*	*p*
Age, time 1 (years)	63.7	6.24	15	61.0	7.55	11	0.97	>0.1
Education (years)	16.2	3.08	15	15.0	2.19	11	1.16	>0.1
Disease duration (years)	3.7	2.18	15	2.9	1.65	11	1.07	>0.1
FTLD-CDR SoB time 1	8.5	2.61	13	8.7	2.90	9	0.18	>0.1
FTLD-CDR SoB time 2	10.2	3.47	13	11.7	2.78	9	1.13	>0.1
MMSE time 1 (total = 30)	25.4	2.73	14	26.1	3.53	11	0.57	>0.1
MMSE time 2 (total = 30)	24.3	4.07	15	19.9	7.70	10	0.87	>0.1
Apathy time 1 (max = 3)	2.0	1.10	11	2.7	1.41	9	1.16	>0.1
Apathy time 2 (max = 3)	1.4	0.97	10	2.7	1.00	9	2.80*	0.012
Disinhibition time 1 (max = 3)	2.2	0.75	11	2.0	1.00	9	0.45	>0.1
Disinhibition time 2 (max = 3)	1.8	0.92	10	1.9	1.17	9	0.18	>0.1
Language time 1 (total = 19)	15.5	2.79	10	8.1	2.84	8	5.51*	0.001
Language time 2 (total = 19)	14.1	3.6	8	7.0	4.27	8	3.63*	0.003
PPT time 1 (total = 26)	23.9	1.86	7	17.9	3.98	7	3.61*	0.006
PPT time 2 (total = 26)	22.1	2.64	8	17.0	3.58	6	2.96*	0.016
BNT time 1 (total = 30)	24.5	3.62	6	6.3	6.34	4	5.21*	0.005
BNT time 2 (total = 30)	22.1	6.38	8	6.3	4.35	4	5.07*	0.001

Mean (M), SD, sample size (*n*), *t* scores, and *p* values of comparisons. Asterisks indicate significant contrasts (*p* < 0.05). Mann–Whitney–Wilcoxon tests were used to confirm results of non-parametric data for disease duration, FTLD-CDR, MMSE, disinhibition, and language.

Disease duration was measured from onset of first reported symptom until time of test at baseline (time 1) and follow-up (time 2). Patients were native speakers of English, and matched for age, education, FTLD clinical dementia rating sum of boxes (FTLD-CDR SoB; Knopman et al., 2008), mini-mental state examination (MMSE; Folstein et al., 1975), and disease duration at time 1 (Table 1), determined by independent *t* tests. Where Shapiro–Wilks tests revealed that demographic data were not normally distributed (bvFTD CDR time 1, *p* = 0.04; svPPA MMSE, *p* = 0.04; svPPA disease duration *p <* 0.01), Mann–Whitney–Wilcoxon tests were used; results did not change (all *p* > 0.1), showing that groups are matched. In addition, repeated measures ANOVAs of FTLD-CDR SoB and MMSE revealed a main effect of testing session (FTLD-CDR SoB: *F*_(20)_ = 9.61, *p* < 0.01; MMSE: *F*_(22)_ = 10.65, *p* < 0.01), but no main effects of group (all *p* > 0.1) or group X session interactions (all *p* > 0.1). We were able to administer abbreviated versions of pyramids and palm trees (PPT; Howard and Patterson, 1992) and Boston naming test (BNT; Kaplan et al., 2001) to a subset of patients within two months of testing at baseline, and within five months at follow-up. Repeated measures ANOVAs of these semantic assessments showed a main effect of group (BNT: *F*_(10)_ = 30.41, *p* < 0.001; PPT *F*_(15)_ = 15.86, *p* < 0.01), but no effects of testing session (all *p* > 0.1) or a group X session interactions (all *p* > 0.1), confirming that svPPA patients were significantly more semantically impaired than bvFTD patients (Table 1).

### Cookie Theft picture description task and analysis

To obtain speech samples, subjects were asked to verbally describe the Cookie Theft picture (Goodglass et al., 1983). Each svPPA and bvFTD subject underwent two recording sessions (time 1 and time 2), with a minimum of six months between testing sessions and an average of 16 months (SD = 6.1). Descriptions were recorded digitally, transcribed, and coded. The total number of nouns produced was tallied for each subject. At time 1, bvFTD patients produced an average of 19.7 nouns (SD = 8.9), svPPA an average of 20.0 nouns (SD = 9.3). At time 2, bvFTD patients produced an average of 14.1 nouns (SD = 7.6), svPPA an average of 15.3 nouns (SD = 12.6). A repeated measures ANOVA revealed a main effect of testing session for both groups (*F*_(24)_ = 8.77, *p* < 0.01), but no effect of group or group X session interaction (*p* > 0.1). The number of nouns per 100 words was also calculated; at time 1, bvFTD patients produced 18.9 nouns per 100 words (SD = 4.3), svPPA an average of 15.4 nouns (SD = 6.0). At time 2, bvFTD patients produced an average of 19.2 nouns per 100 words (SD = 6.1), and svPPA an average of 16.4 nouns (SD = 3.8). A repeated measures ANOVA revealed no effect of testing session (*p* > 0.1), group (*p* = 0.08), or group X session interaction (*p* > 0.1). These results indicate that total noun production did not significantly differ between svPPA and bvFTD patients, although it did decline by the second testing session.

The concreteness of each noun was rated based on published norms (Brysbaert et al., 2014), on a scale from 1 (abstract) to 5 (concrete); thus declining concreteness scores are equivalent to increasing abstractness scores. Examples of concreteness of nouns produced by patients are way (2.34), distraction (2.46), time (3.07), stuff (3.13), story (3.3), day (3.92), home (4.11), mother (4.6), children (4.78), stool (4.9), cookie (5), and water (5). The average concreteness of nouns produced was calculated for each subject. In addition, to minimize the potential bias associated with repetition of a particular word type, we tabulated each unique instance of a noun, and averaged the concreteness for unique nouns produced. To examine how concreteness changes over time, linear mixed effects models were performed using the package lmerTest in R. To compare models, restricted maximum likelihood (REML) was not used. Linear mixed effects models assessed change in the concreteness of the nouns that were produced as a function of disease duration at times 1 and 2 and differences between patient groups. By including disease duration as a factor, we were able to account for differences between testing sessions. Individual was included as a random effect. Education was included as a fixed effect because more years of education is significantly associated with more abstract speech for both svPPA (ρ_(18)_ = –0.68, *p* < 0.001) and bvFTD (ρ_(26)_ = –0.38, *p* = 0.038) patients. In sum, to test for an interaction between patient group and longitudinal change in concreteness we used the formula: lmer(concreteness ∼ disease_duration * patient_group + education + (1|individual). An ANOVA compared this interaction model to a null model, which did not include an interaction between longitudinal change and patient group.

### Imaging collection and analysis

Initial T1-weighted structural images were collected on 10 of 11 svPPA patients on a Siemens 3T scanner using an 8-channel head coil with repetition time = 1620 ms, echo time = 3 ms, flip angle = 15°, matrix = 192 × 256, slice thickness = 1 mm, and in-plane resolution = 0.9 × 0.9 mm. There was an average of 2.6 months (SD = 3.1) between Cookie Theft test and MRI acquisition at time 1. For a baseline comparison, images were collected on an independent group of age- and education-matched elderly controls (*n* = 21). Images for this cross-sectional comparison and for the regression analyses (described below), were processed with antsCorticalThickness, built on state-of-the-art Advanced Normalization Tools (ANTs; https://github.com/ANTsX/ANTs; Tustison et al., 2014). Longitudinal structural images were available for a subset of svPPA patients (*n* = 7), with an average of 3.6 months (SD = 1.1) between the Cookie Theft recording and MRI acquisition at time 2. Longitudinal images were preprocessed using the antsLongitudinalCorticalThickness pipeline (Tustison et al., 2018). For each patient, time 1 and time 2 images were used to create a single subject template, which was then used to process the individual scans. GM images were normalized to Montreal Neurologic Institute (MNI) space, segmented, down-sampled to 2 mm^3^ resolution, and smoothed using a 2-mm full-width half-maximum (FWHM) Gaussian kernel to maintain sensitivity to typical human cortical thickness. To calculate the annualized change in GM for each subject, GM volume at time 2 was subtracted from volume at time 1; GM change was normalized by the time in years between scans.

Preprocessed images were analyzed using FSL’s Randomise tool to perform nonparametric permutation analyses with 10 000 permutations. To identify regions of significant atrophy at time 1 in svPPA, we compared GM to controls using a two-sample *t* test with a threshold of *p* < 0.05, family-wise error corrected with threshold free cluster enhancement. To identify regions of significant longitudinal change in cortical thickness, a one-sample *t* test was performed on the annualized GM change images (*n* = 7), using a height threshold of *p* < 0.05 (uncorrected) and minimum cluster size of 50 voxels. Voxelwise multiple regression related annualized GM change to the annualized change in noun concreteness in svPPA, height threshold of *p* < 0.01 (uncorrected) and minimum cluster size of 15 voxels. This analysis may not capture regions that are already severely atrophied at time 1 and show only minimal changes at time 2. Thus, GM thickness at time 1 was also related to annualized change in concreteness (*n* = 10), constrained to regions of significant atrophy. These analyses are uncorrected for multiple comparisons and use a liberal threshold because we use a high-resolution voxel size of 2 mm^3^ that reflects cortical anatomy and because our longitudinal sample size is relatively small due to the rarity of svPPA (Landin-Romero et al., 2016).

## Results

### Changes in concreteness

**Figure 1.**
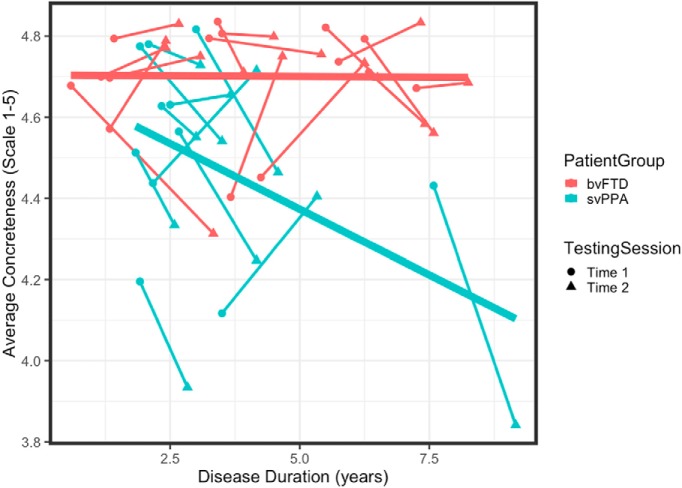
Longitudinal change in concreteness of nouns produced by patients. The average concreteness for each individual with bvFTD (red) and svPPA (blue) at Time 1 (circle) and Time 2 (triangle) by disease duration at Time 1 and 2. Testing sessions for each individual are connected by broken line. Nouns were scored on a scale of abstract (1) to concrete (5) based on published norms. Bold trend lines plot the linear model between Concreteness and Disease Duration for each patient group.

Speech samples produced by both bvFTD and svPPA patients were highly concrete at baseline, with 20 of 26 patients scoring >4.5 out of a possible 5 at time 1 (Fig. 1). However, a one-way ANCOVA covarying for education (*F*_(23)_ = 4.29, *p* < 0.05) shows that the average noun concreteness for svPPA (M = 4.54, SD = 0.23) is significantly less concrete than bvFTD patients (M = 4.70, SD = 0.13) at baseline (time 1; *F*_(23)_ = 5.96, *p* < 0.05). To examine how concreteness changes with time, Figure 1 plots longitudinal change in the average concreteness of nouns produced with disease duration at times 1 and 2 in svPPA and bvFTD. We tested this relationship in each patient group using a linear mixed-effects model that included education as a fixed effect and individual as a random effect. In svPPA, disease duration (β = –0.05, *t*_(15)_ = 2.26, *p* < 0.05) and education (β = –0.07, *t*_(15)_ = –3.34, *p* < 0.01) significantly predicted declines in average concreteness in svPPA (marginal *r*^2^ = 0.48, conditional *r*^2^ = 0.49). Cook’s distance was calculated to identify any points with high leverage. One svPPA patient was revealed to be highly influential in our model (Cook’s D = 0.95) compared to other subjects (Cook’s D < 0.2), however results did not change and remained significant after removing this patient. A mixed-effects model in bvFTD revealed no longitudinal change in the average concreteness of nouns produced with increasing disease duration (*p* > 0.1).

We next tested whether change in concreteness was significantly different across svPPA and bvFTD patients (Table 2, interaction model). A mixed-effects model revealed a significant interaction between disease duration and patient group (*t*_(35.7)_ = 3.11, *p* < 0.01). In addition, individuals with higher education were found to produce more abstract nouns (*t*_(27.3)_ = 3.10, *p* < 0.01). Overall, this model had good fit, explaining a moderate proportion of the variance observed in noun concreteness (marginal *r* ^2^ = 0.48; conditional *r* ^2^ = 0.52). We compared this interaction model (disease duration X group) to a null model (group) with no interaction (Table 2). An ANOVA comparing the null model and the interaction model (Table 2) showed that including disease duration X group interaction significantly improved model fit (χ^2^_(1)_ = 8.57, *p* < 0.01), indicating that declining noun concreteness over time in svPPA was significantly different from in bvFTD. Finally, to ensure that repetition of highly concrete or abstract nouns was not driving the concreteness dissociation between svPPA and bvFTD, we examined longitudinal changes in the average concreteness of unique nouns produced. Again, we found a significant interaction between disease duration and patient group for the concreteness of unique nouns produced (β = 0.04, *t*_(52)_ = 2.14, *p* < 0.05), with a high proportion of variance explained by fixed effects (marginal *r* ^2^ = 0.44).

**Table 2.** Results for mixed effects model of average concreteness (null and interaction) and ANOVA comparing the two

**Interaction model**		**Fixed effects**				**Random effects**		
		**β**	**SE**	***t***	***p***		**Variance**	**SD**
bvFTD Intercept		5.116	0.150	34.08	<0.001	**Individual**	0.002	0.048
Disease duration		0.019	0.016	1.199	0.239	**Residual ε**	0.025	0.158
svPPA		0.022	0.106	0.203	0.841			
Education		–0.031	0.010	–3.10	0.004			
Disease Duration*svPPA		–0.078	0.025	–3.11	0.004			
**Null model**		**Fixed effects**				**Random effects**		
		**β**	**SE**	***t***	***p***		**Variance**	**SD**
bvFTD intercept		5.153	0.172	30.01	<0.001	**Individual**	0.006	0.079
Disease duration		–0.011	0.014	–0.792	0.433	**Residual ε**	0.026	0.162
svPPA		–0.272	0.057	–4.77	<0.001			
Education		–0.025	0.011	–2.23	0.034			
**ANOVA**	**df**	**AIC**	**BIC**	**Log likelihood**	**deviance**	**χ^2^**	**df**	***p***
Interaction model	7	–26.20	–12.54	20.10	–40.20	8.57	1	0.003
Null model	6	–19.62	–7.91	15.81	–31.62			

To create an index of change for each patient, we calculated the annualized change in concreteness: concreteness at time 1 was subtracted from concreteness at time 2, normalized by the time in years between testing sessions. Annualized change in concreteness was greater for svPPA (M= –0.124, SD = 0.179) than bvFTD patients (M = 0.002, SD = 0.155). A one-way ANCOVA revealed a significant difference in annualized change between groups (*F*_(22)_ = 4.64, *p* < 0.05), controlling for baseline concreteness (*F*_(22)_ = 4.73, *p* < 0.05) and education (*F*_(22)_ = 3.34, *p* = 0.08).

### Structural imaging in svPPA

**Figure 2.**
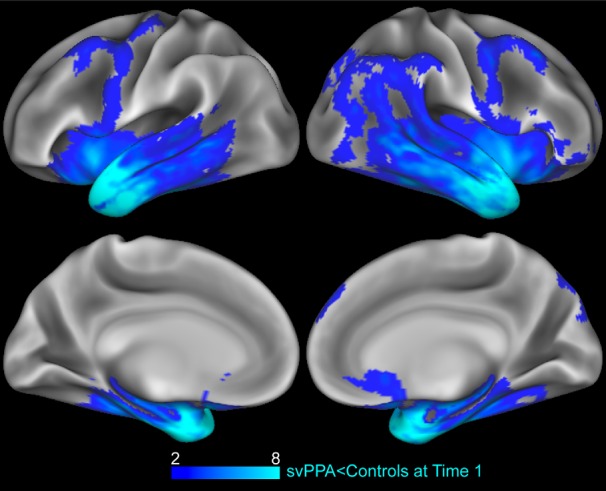
Regions of atrophy in svPPA at baseline. Regions of GM that are significantly reduced in svPPA at Time 1 compared to elderly controls (blue). Heat map intensity refers to t-values.

**Figure 3.**
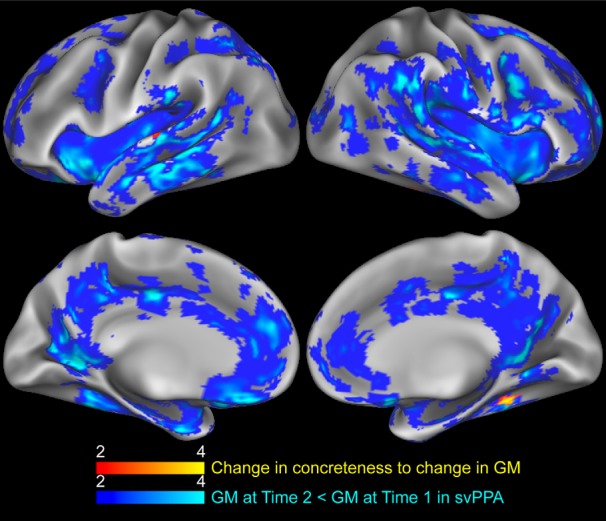
Longitudinal changes in GM and noun concreteness in svPPA. Regression of annualized change in concreteness to annualized GM change in svPPA (red). Regions of GM that are significantly reduced in svPPA at Time 2 compared to Time 1 (blue). Heat map intensity refers to t-values.

A two-sample *t* test identified regions of significant atrophy in svPPA at time 1, compared to controls, revealing extensive and severe atrophy in bilateral superior, middle and inferior anterior temporal lobes (Table 3). Significant atrophy also includes bilateral inferior frontal lobes, posterior temporal lobes, and right superior, middle, inferior and medial frontal, and right parietal cortex (Fig. 2, blue). We next evaluated the annualized change in cortical thickness (Fig. 3, blue). A one-sample *t* test revealed that svPPA patients had severe bilateral GM declines in medial and lateral regions of the temporal lobe, and to the inferior frontal lobes (Fig. 2; Table 4). Declines in annualized GM extend caudally to medial parietal regions, including cingulate and precuneus, and right occipital regions, and are more extensive in the right hemisphere. Atrophy also extends dorsally to middle and superior frontal regions, especially to left medial frontal regions including the left anterior cingulate. Tables 3, 4 index peak-voxel coordinates and cluster extent.

**Table 3.** Summary of regions of reduced GM at time 1 in svPPA, compared to controls

svPPA-GM < control-GM at time 1	BA	*x*	*y*	*z*	k	*t* score
Left superior temporal	38	–22	12	**–**42	13670	14.3
Left parahippocampal	36	**–**30	–8	**–**34	Sub	14
Left parahippocampal	36	–24	–6	**–**46	Sub	13.2
Left parahippocampal	36	–22	–6	**–**36	Sub	13.1
Left inferior temporal	20	**–**34	–2	**–**46	Sub	13.1
Right superior temporal	38	40	24	**–**38	19037	14.3
Right superior temporal	38	26	20	**–**36	Sub	12.8
Right inferior temporal	20	56	–6	**–**32	Sub	12.6
Right middle temporal	21	44	16	**–**40	Sub	12.3
Right inferior temporal	20	26	0	**–**40	Sub	11.9
Right superior frontal	8	10	48	48	169	4.63
Right middle frontal	10	26	62	22	18	4.26
Right middle frontal	10	34	64	4	44	3.74
Right superior frontal	10	26	54	**–**4	16	3.33
Right middle frontal	11	40	50	–10	134	3.32
Right inferior frontal	45	52	26	8	24	2.83

Brodmann area (BA), coordinates (*x*, *y*, *z*) of peak-voxels, cluster extent (k), subpeaks for cluster extent >200 (sub), and *t* score are provided for each cluster.

**Table 4.** Summary of annualized GM decline in svPPA

Time 2 GM < time 1 GM in svPPA	BA	*x*	*y*	*z*	k	*t* score
Left posterior cingulate	30	–18	–52	4	24770	14.9
Left posterior cingulate	30	–18	–64	8	Sub	13.6
Left superior temporal	22	–66	–24	2	Sub	13.1
Right inferior frontal	44	58	14	14	Sub	10.7
Left medial frontal	9	–4	48	18	Sub	10.6
Left superior temporal	39	–42	–56	28	70	8.97
Left inferior parietal	39	–38	–70	42	60	5.95
Left premotor	6	–26	0	64	109	5.66
Left superior parietal	7	**–**32	**–**58	66	806	5.42
Left superior parietal	7	–26	**–**56	48	Sub	4.69
Left inferior parietal	40	**–**44	**–**44	54	Sub	4.53
Left inferior parietal	40	**–**42	**–**48	42	Sub	4.46
Left superior parietal	7	–26	**–**52	50	Sub	4.38
Left middle occipital	19	–26	–90	14	61	5.4
Right middle occipital	19	44	–84	16	177	5.06
Left superior frontal	6	–6	–2	74	91	4.76
Left inferior frontal	44	–52	12	28	360	3.89
Left precentral	9	**–**36	4	40	Sub	3.48
Left inferior frontal	44	**–**42	8	20	Sub	3
Left middle frontal	9	**–**36	12	26	Sub	2.85
Left inferior frontal	44	–58	18	26	Sub	2.8
Left middle frontal	8	–28	22	46	758	3.54
Left superior frontal	8	**–**4	40	54	Sub	3.48
Left superior frontal	8	**–**30	26	56	Sub	3.38
Left superior frontal	8	**–**32	26	52	Sub	3.14
Left middle frontal	8	**–**34	34	42	Sub	2.85
Left postcentral	7	–22	**–**48	70	57	3.1
Left middle frontal	6	–26	–6	50	141	2.76
Left middle frontal	9	**–**40	44	30	51	2.63
Left inferior parietal	40	42	–52	44	64	2.45

Brodmann area (BA), coordinates (*x*, *y*, *z*) of peak-voxels, cluster extent (k), subpeaks for cluster extent >200 (sub), and *t* score.

We used the annualized change in concreteness index for each svPPA patient to test how changes in GM relate to the concreteness of nouns produced. A regression analysis revealed that annualized decline in concreteness was related to annualized GM decline in right fusiform and parahippocampal gyri and the left superior temporal gyrus (Fig. 3; Table 5). At height threshold of *p* < 0.01, no significant clusters related to increases in concreteness in svPPA. At a less conservative threshold of *p* < 0.05, we found that declining GM in right inferior frontal (MNIxyz = 48, 22, 6; k = 35; *t* = 4.29), right middle occipital (MNIxyz = 32, –82, 22; k = 17; *t* = 6.19), and left superior temporal regions (MNIxyz = –66, –46, 14; k = 31; *t* = 6.04). Finally, to test whether atrophy at time 1 predicts declining concreteness, we related GM at time 1 in svPPA to subsequent annualized decline in concreteness. At height threshold of *p* < 0.01, no significant clusters were related GM at time 1 to decline in concreteness in svPPA. At a less conservative threshold of p < 0.05, analyses reveal that decreased GM of the right parahippocampus (MNIxyz = 36, 24, –8; k = 21; *t* = 2.89) and right insula (MNIxyz = 32, 16, 0; k = 31; *t* = 4.42) at time 1 was associated with a steeper decline in noun concreteness.

**Table 5.** Regression of annualized decline in GM to annualized decline in concreteness

Decline in GM and concreteness	BA	*x*	*y*	*z*	k	*t* score
Right parahippocampal	36	30	**–**42	–8	27	6.04
Left superior temporal	42	–62	–20	6	6	5.5
Right fusiform	37	50	**–**44	–24	18	5.34

Brodmann area (BA), coordinates (*x*, *y*, *z*) of peak-voxels, cluster extent (k), and *t* score are provided for each cluster.

## Discussion

This study examines longitudinal change in the production of concrete nouns in svPPA compared to bvFTD patients. While speech samples produced by both bvFTD and svPPA patients were highly concrete at baseline, svPPA patients produced significantly less concrete nouns than bvFTD patients. Mixed-effect models revealed a significant decrease in concreteness of nouns produced with disease progression in svPPA patients, while noun concreteness in bvFTD remained high for both testing sessions. Longitudinal declining concreteness in svPPA was associated with progressive GM atrophy in right ventral and left lateral temporal regions. Results also revealed that higher educational attainment is associated with more abstract noun production for both bvFTD and svPPA patients. The declining concrete noun production in svPPA we observe here and observed by (Hoffman et al., 2012) may explain, in part, the variation in reversal of CE observed across different cross-sectional studies (Breedin et al., 1994; Bonner et al., 2009; Jefferies et al., 2009; Hoffman et al., 2013; Joubert et al., 2017). If concrete knowledge tends to decline more rapidly than abstract knowledge in svPPA, this can provide important prognostic information to patients and caregivers who must develop strategies for communication in the face of progressive semantic impairment.

The annualized decrease in noun concreteness in svPPA was associated with longitudinal GM atrophic progression of the right ventral temporal lobe and left superior temporal gyrus. The bilateral ventral temporal lobe, including the parahippocampal and fusiform gyri, is a portion of visual association cortex involved in object feature representation, recognition and discrimination (Lambon Ralph et al., 2010; Mion et al., 2010; Clarke and Tyler, 2015; Bi et al., 2016; Bonner et al., 2016; Ding et al., 2016; Shebani et al., 2017). A gradient of informational specificity and complexity may organize the ventral visual pathway (Bonner and Price, 2013; Clarke and Tyler, 2014), with elementary visual-perceptual features associated with coarse categorical representations in posterior regions, and discrimination of highly confusable words associated with overlapping and more meaningful visual features in anterior portions, including the perirhinal cortex (Inhoff and Ranganath, 2015; Wright et al., 2015; Martin et al., 2018). Concrete nouns refer to tangible objects that are highly associated with visual features, and our results support the hypothesis that degradation of visual feature concepts due to the ventral and middle temporal lobe atrophy in svPPA impairs knowledge of concrete nouns, including the production of concrete nouns in speech. Progressive atrophy of the left superior temporal gyrus was associated with declining concrete noun production, and at a more lenient threshold, also with declining abstract noun production. The left superior posterior temporal lobe has been associated with lexical retrieval and production (Indefrey, 2011; Geranmayeh et al., 2015) and its involvement here is likely related to task demands during picture description. At the more lenient threshold, the annualized decrease in noun concreteness in svPPA was associated with atrophy of the right parahippocampus and insula at time 1. While we do not have strong hypotheses regarding the role of the right insula in concrete noun production, one study has linked the bilateral insula to the processing of semantically precise words (Chan et al., 2004), compared to semantically ambiguous words which others have shown tend to be more abstract (Hoffman et al., 2012). In sum, our findings indicate that the reversal of CE becomes more severe with time in svPPA and that this is related to progressive atrophy within the right ventral temporal lobe. This longitudinal work emphasizes the link between degraded concrete noun knowledge and progressive atrophy of visual association cortex that is important for representing visual feature knowledge about concrete object concepts.

Our findings are in accordance with a longitudinal behavioral study of word naming in svPPA by Hoffman and colleagues (Hoffman et al., 2012). Their study examined words with high and low visual content, a feature that is highly correlated with concreteness. They found that naming for high and low visual items was equivalent at baseline. However, as disease severity increased, svPPA patients had increasing difficulty naming highly visual items compared to items with low visual content, showing reversal of CE. This is despite the finding that highly visual items were easier to name than low visual items in healthy adults. Hoffman and colleagues interpreted their findings according to the Hub-and-Spoke model of conceptual knowledge (Patterson et al., 2007). Theoretically, the initial equivalent naming for high and low visual items is because disease in svPPA originates in the anterior temporal lobe, or a pan-model hub of conceptual knowledge. They hypothesized that, with increasing atrophy within the ventral temporal lobe, or “visual spoke” of conceptual knowledge, knowledge for highly visual items declines in svPPA, giving rise to reversal of CE. Our study provides anatomic evidence that supports this hypothesis; we see that progressive atrophy to the middle and ventral temporal lobe in svPPA is associated with declining noun concreteness.

Not all studies show an increased reversal of CE in svPPA over time. Macoir (2009) compared concrete and abstract word knowledge in one svPPA patient over 21 months. For this patient, the reversal of CE disappeared with time, leading to an equivalent impairment for concrete and abstract words at follow-up. One source of disparity between these findings may be differences in disease severity at the time of observation. In another longitudinal examination of both semantic impairment and atrophy spread in svPPA, two patients were followed over a three-year period (Bright et al., 2008). The study showed that poor comprehension of object concepts was linked to atrophy of the inferior temporal gyrus at time 1. As atrophy spread posteriorly to the posterior superior temporal lobe and dorsally to the frontal lobe, patients demonstrated more generalized language impairments. While the study did not examine abstract word knowledge, at the time of final testing, patients’ disease encompassed regions shown to be important for abstract concept processing, including left inferior frontal gyrus and superior temporal lobe (Sabsevitz et al., 2005; Wang et al., 2010). In the present study we observe that GM of the right inferior frontal lobe in svPPA declines between times 1 and 2, and that emerging declines in noun abstractness were related to progressive atrophy in right inferior frontal and left superior temporal regions. Although this relationship was only detectable at a lenient height threshold, we might predict that svPPA patients would show an accelerated loss of abstract concepts as disease continues to accumulate in the inferior frontal lobe.

Cognitive and anatomic longitudinal studies of svPPA are rare, and none have examined how GM disease progression relates to declining concrete noun knowledge. This study fills an important gap in our understanding of how changes of focal atrophy in svPPA relate to reversal of CE. However, important caveats to these findings are that we do not compare production to healthy controls, and that we were able to obtain longitudinal imaging on only a small number of patients due in part to the rarity of svPPA, although this number is relatively large compared to other longitudinal studies. Another limitation is that Cookie Theft speech samples contain highly concrete content due to the visual presentation of the picture guiding the semi-structured speech sample. Since many nouns used to describe the scene were close to a maximum value of 5, our ability to observe increases in the concreteness of speech over time was restricted. This may have impacted our ability to detect an abstract semantic impairment in bvFTD patients (Cousins et al., 2016, 2017). Despite this, there was a significant difference in annualized change between svPPA and bvFTD; svPPA patients declined significantly in the concreteness of nouns produced, while patients with bvFTD maintained highly concrete noun production throughout the course of testing. Future studies should examine whether this decline is due to omission or neglect of concrete material in the scene, or due to use of substitutions that are more abstract. Finally, our study is a snapshot within the disease course of svPPA. On average, our patients had been living with symptoms for 2.8 years at the time of initial testing (disease duration at time 1), and we measured change over an average of 16 months (time 2). As revealed by our longitudinal structural imaging analyses, GM loss in svPPA is most apparent in left and right ventral and lateral mid-temporal regions, and this atrophy is related to their decreased production of concrete nouns. In addition, we observed GM decline in the frontal lobe, including the inferior frontal gyrus, which has been previously linked to abstract noun knowledge and processing (Sabsevitz et al., 2005; Hoffman et al., 2015; Cousins and Grossman, 2017). It may be that as disease progresses and encompasses frontal regions, abstract noun processing would become increasingly impaired in svPPA. Future experiments should examine how changes in the concreteness of speech proceed with time and progressive degeneration.

## Synthesis

Reviewing Editor: Morgan Barense, University of Toronto Decisions are customarily a result of the Reviewing Editor and the peer reviewers coming together and discussing their recommendations until a consensus is reached. When revisions are invited, a fact-based synthesis statement explaining their decision and outlining what is needed to prepare a revision will be listed below. The following reviewer(s) agreed to reveal their identity: Chris Martin, Siddharth Ramanan.

We all agreed that the resubmission does a terrific job in addressing concerns raised in the previous round of reviews. Only three minor outstanding issues remain:

1. The O'Connor (2017) reference appears incomplete in the references section.

2. Page 5, line 87 - ‘bvFTD patients have relatively spared temporal lobe integrity’ - the authors have not clarified if they mean relatively preserved integrity of the lateral or medial temporal lobes.

3. There appears to be some sort of artefact between the color bars in Figures 2 and 3.
